# CK2 Phosphorylation of *Schistosoma mansoni* HMGB1 Protein Regulates Its Cellular Traffic and Secretion but Not Its DNA Transactions

**DOI:** 10.1371/journal.pone.0023572

**Published:** 2011-08-24

**Authors:** Isabel Caetano de Abreu da Silva, Vitor Coutinho Carneiro, Renata de Moraes Maciel, Rodrigo Furtado Madeiro da Costa, Daniel Rodrigues Furtado, Francisco Meirelles Bastos de Oliveira, Mário Alberto Cardoso da Silva-Neto, Franklin David Rumjanek, Marcelo Rosado Fantappié

**Affiliations:** Instituto de Bioquímica Médica, Programa de Biologia Molecular e Biotecnologia, Universidade Federal do Rio de Janeiro, Ilha do Fundão, Rio de Janeiro, Brasil; Fundação Oswaldo Cruz, Brazil

## Abstract

**Background:**

The helminth *Schistosoma mansoni* parasite resides in mesenteric veins where fecundated female worms lay hundred of eggs daily. Some of the egg antigens are trapped in the liver and induce a vigorous granulomatous response. High Mobility Group Box 1 (HMGB1), a nuclear factor, can also be secreted and act as a cytokine. Schistosome HMGB1 (SmHMGB1) is secreted by the eggs and stimulate the production of key cytokines involved in the pathology of schistosomiasis. Thus, understanding the mechanism of SmHMGB1 release becomes mandatory. Here, we addressed the question of how the nuclear SmHMGB1 can reach the extracellular space.

**Principal Findings:**

We showed *in vitro* and *in vivo* that CK2 phosphorylation was involved in the nucleocytoplasmic shuttling of SmHMGB1. By site-directed mutagenesis we mapped the two serine residues of SmHMGB1 that were phosphorylated by CK2. By DNA bending and supercoiling assays we showed that CK2 phosphorylation of SmHMGB1 had no effect in the DNA binding activities of the protein. We showed by electron microscopy, as well as by cell transfection and fluorescence microscopy that SmHMGB1 was present in the nucleus and cytoplasm of adult schistosomes and mammalian cells. In addition, we showed that treatments of the cells with either a phosphatase or a CK2 inhibitor were able to enhance or block, respectively, the cellular traffic of SmHMGB1. Importantly, we showed by confocal microscopy and biochemically that SmHMGB1 is significantly secreted by *S. mansoni* eggs of infected animals and that SmHMGB1 that were localized in the periovular schistosomotic granuloma were phosphorylated.

**Conclusions:**

We showed that secretion of SmHMGB1 is regulated by phosphorylation. Moreover, our results suggest that egg-secreted SmHMGB1 may represent a new egg antigen. Therefore, the identification of drugs that specifically target phosphorylation of SmHMGB1 might block its secretion and interfere with the pathogenesis of schistosomiasis.

## Introduction

Schistosomes are parasitic blood flukes infecting approximately 200 million people globally [1]. *Schistosoma mansoni* parasites reside in mesenteric veins, where they lay hundreds of eggs per day, 4–5 weeks post-infection. After initial infection, larval and adult parasites produce minimal inflammatory pathology in the host. However, by the time the eggs are laid, some of them are trapped in the microvasculature of the liver causing the granuloma, due to a periovular inflammatory reaction. Granulomas are initially macrophage reactions of the foreign body type, essentially mobilizing the circulating monocytes. Following maturation of the embryo (miracidium) and secretion of potent soluble egg antigens, the T-lymphocyte circuits elicit an inflammatory reaction promoting cellular recruitment and activation, which are dependent upon the local production of a vast array of cytokines [2]–[4]. The pre-postural phase of schistosomal infection is characterized by a Th1 dominant reaction. After the beginning of oviposition, the egg-derived antigens elicit a strong Th2 reaction with high levels of IL-4 and IL-5 [2]. The intensity of the granulomatous reaction peaks in mice from the 7^th^ week onwards, but subsequently the inflammation reaction is down-modulated despite the continuous production of adult worm and egg-derived antigens. Much of the morbidity of schistosomiasis is attributed to the egg-induced granulomatous responses, particularly to the fibrosis associated with it, which is thought to be associated with periportal hypertension. Although Praziquantel is highly effective in curing *S. mansoni* infection, liver granulomas persist for life, as the eggs cannot be eliminated. Thus, chemotherapy to prevent the morbidity associated with liver egg granulomas would represent a major improvement in the pathology of schistosomiasis.

High Mobility Group Box 1 (HMGB1) is a highly conserved component of eukaryotic nuclei [5]–[6]. HMGB1 is ubiquitous and only slightly less abundant than core histones. It has a tripartite structure, composed of two homologous DNA-binding domains, the A and B HMG-boxes, and a C-terminal acidic domain [5]. HMGB1 is located in the nucleus, where it acts as an architectural protein that can promote DNA bending, supercoiling and unwinding. These DNA transactions performed by HMGB1 promote the assembly of site-specific DNA-binding factors, and are involved in transcription [7]. The phenotype of *Hmgb1* knockout mice confirmed the functional importance of HMGB1 as a regulator of transcription: they die shortly after birth showing a defect in transcriptional control exerted by the glucocorticoid receptor [8].

In addition to transcriptional regulation, HMGB1 has extracellular roles. In 1999, during a course of experiments designed to identify late-acting mediators of endotoxaemia and sepsis, it was discovered [9] that activated macrophages secrete HMGB1 as a delayed mediator of inflammation. HMGB1 is regarded as a prototypic alarmin, a kind of endogenous danger-associated molecular patter (DAMP), as it is released by necrotic (but not apoptotic) cells or secreted by immune cells in response to tissue damage [10]. Activated macrophages secrete HMGB1 as a delayed mediator of inflammation, well after peak of TNF-α and IL-1 [9]. HMGB1 promotes monocytes recruitment and release of pro-inflammatory cytokines such as TNF-α, IL-1, IL-6 and IL-8 [9]–[12], signaling through RAGE (receptor for advanced glycation end-products) [13], toll-like receptor 2 (TLR2) and TLR4 [14]–[15]. The delayed HMGB1 release can have lethal consequences in sepsis, as administration of HMGB1-specific antibodies confers significant protection against mortality in endotoxaemia [10]. Thus, secreted HMGB1 also functions as an inflammatory cytokine, and its secretion is pivotal in sepsis. Nevertheless, the exact mechanism(s) that control(s) its secretion is still poorly understood.

Recent studies have shown that the post-translational modification status of mammalian HMGB1 (mHMGB1) is related to its translocation within cells and secretion by inflammatory cells, in which it shuttles between the nucleus and cytoplasm through a process that involves the HMGB1 hyperacetylation [6]. Cytosolic mHMGB1 accumulates and is secreted through a vesicle-mediated secretory pathway [6]. More recently however, it has been reported that phosphorylation [16]–[19], methylation [20] and poly (ADP) ribosylation [21] also play a role in the cellular traffic of mHMGB1.

Along with histone H1, HMGB1 protein appear to be among the most highly phosphorylated protein species in the nucleus [22]. Phosphorylation of HMGB1 proteins from plants and insects has been reported to modulate their stability and DNA binding activities [23]–[25]. For example, phosphorylation of maize HMGB1 by CK2 increases the negative net charge of the acidic tail, which strengthens electrostatic interactions with the HMG-box basic domains. Accordingly, phosphorylation reduced the affinity of maize HMGB1 for linear DNA [24]. Similarly, phosphorylation of insect HMGB1 proteins by PKC resulted in a tenfold reduction of their DNA binding strength [25]. For the mammalian HMGB1 protein, the role of phosphorylation on its DNA binding activities has not yet been well documented. On the other hand, it has been recently reported that PKC and CaMK phosphorylation plays an important role in the nucleocytoplasmic transport of mHMGB1 [16]–[19]. It was shown that the shuttling of mHMGB1 between the nucleus and cytoplasm is tightly controlled by the phosphorylation of the two nuclear localization signals (NLS) of mHMGB1 [20].

We have previously cloned the *S. mansoni* HMGB1 cDNA and have fully characterized its DNA-related activities [26]. SmHMGB1 showed a high degree of conservation among the HMG box domains when compared to its mammalian counterpart [26]. However, SmHMGB1 differed significantly in its C-terminal acidic tail, consisting of only five acidic residues (mHMGB1 contains 30 continuous acidic residues in its tail). Results from Gnanasekar *et al* [27] investigating the pro-inflammatory activities of SmHMGB1, showed *in vitro* that significant levels of SmHMGB1 were present in excretory secretions of eggs. They also showed that SmHMGB1 was a potent inducer of pro-inflammatory cytokines such as TNF-α, IL-1Rα, IL-2Rα, IL-6, IL-13, IL-13α1, IL-15 and MIP-1α from peritoneal macrophages [27]. The TNF-α-inducing effect was a function of the B box domain of SmHMGB1 (similar to the mammalian HMGB1) and this effect could be blocked by neutralizing antibodies against SmHMGB1 [27]. These findings pose SmHMGB1 as a major inflammatory factor among egg excretory secretions and an attractive candidate to be targeted by chemotherapy in schistosomiasis.

Hyperacetylation of SmHMGB1 is important for its exit to the extracellular milieu, as we have recently shown [28]. Here, we showed that besides acetylation, SmHMGB1 is phosphorylated by CK2, PKA and PKC, and provided evidence that phosphorylation by CK2 plays an important role in the translocation of SmHMGB1 from the nucleus to the cytoplasm. However, we demonstrated that phosphorylation of recombinant or endogenous SmHMGB1 did not affect its DNA binding activity. Finally, we showed that SmHMGB1 proteins that were located in the cytoplasm of adult worms, in egg secretions or in the periovular granuloma, were phosphorylated, indicating that phosphorylation actively participates in SmHMGB1 secretion.

In the present work we describe the molecular characterization of the mechanism of SmHMGB1 transfer from the nucleus to the cytoplasm, culminating with its extracellular release. Furthermore, we hypothesize that SmHMGB1 might act as an important immune modulator for the development of the hepatic schistosomotic granuloma.

## Materials and Methods

### Ethics statement

All animals were handled in strict accordance with good animal practice as defined by Animals Use Ethics Committee of UFRJ (Universidade Federal do Rio de Janeiro), with approval ID # IBqM 038. The study was conducted adhering to the institution's guidelines for animal husbandry.

### Plasmids

Complementary DNAs encoding recombinant SmHMGB1-FL, SmHMGB1-ΔC, SmHMGB1-box domain A and SmHMGB1-box domain B were previously described [26]. Complementary DNAs encoding recombinant single or double amino acid mutants (S172A, S174A and S172A/S174A) were amplified by RT-PCR using sense primer F1 (5′-*GGATCC* ATGGCTGAAGACAAGGGTAAG- 3′) (*Bam*HI restriction site is in italic and initiation codon is underlined) and anti-sense primers M1 (5′*AAGCTT* CTAATCGTCAGACTCTGCATCTTC3′) for the SmHMGB1 S172A, M2 (5′*AAGCTT* CTAATCGTCTGCCTCTGAATCTTC3′) for the SmHMGB1 S174A and M3 (5′*AAGCTT* CTAATCGTCTGCCTCTGCATCTTC3′) for the SmHMGB1 S172A/S174A (*Hind*III restriction site is in italic, and the termination codon is underlined). RT-PCR was performed on *S. mansoni* adult worm cDNAs, sub-cloned into pCR2.1 TOPO plasmid (Invitrogen), and sequenced on both strands (Macrogen Inc., Korea). In order to generate recombinant his-tagged proteins, plasmids were digested with the appropriate enzymes (Promega) and cloned into the pQE-80L expression vector (Qiagen), according to the manufacturer's instructions. For EGFP analysis, cDNAs encoding SmHMGB1 full-length (SmHMGB1-FL, aa residues 1–176) or SmHMGB1 mutated at both serines located in its C-terminus (S172A/S174A) were cloned downstream of EGFP in pEGFP-C3 vector (BD Clontech), and these constructs named EGFP-SmHMGB1 and EGFP-SmHMGB1-S172A/S174A, respectively. We generated these constructs by PCR amplifications using *S. mansoni* adult worm cDNAs with the sense primer F2 (5′-*AAGCTT* ATGGCTGAAGACAAGGGTAAG-3′) (*Hind*III restriction site is in italic and initiation codon is underlined) and anti-sense primers F3 (5′GGATCCCTAATCGTCAGACTCTGAATC3′) and M4 (5′*GGATCC* CTAATCGTCTGCCTCTGCATC3′) for the full-length SmHMGB1.

### Expression of recombinant proteins and polyclonal antibody production

Full-length SmHMGB1 (aa residues 1–176), the protein lacking its acidic tail, SmHMGB1-ΔC (aa residues 1–169), domain A only (aa residues 1–83), domain B only (aa residues 84–169) and SmHMGB1 mutants (S172A, S174A and S172A/S174A) were expressed with (His)_6_-tag at their N-termini as previously described [26]. Protein concentration was determined by the Bio-Rad Protein Assay (Bio-Rad). Purity of HMGB1 proteins was checked by 12 or 15% SDS-PAGE, followed by Coomassie Blue R-250 staining. Polyclonal rabbit serum was produced against preparations of recombinant SmHMGB1-box domain B. Rabbits were inoculated with 50 µg of protein mixed with complete Freund's adjuvant (SIGMA) and boosted four times with 50 µg of protein mixed with incomplete Freund's adjuvant (SIGMA). Pre-immune serum was collected before the first immunization.

### Phosphorylation assays

Recombinant SmHMGB1 proteins (1 µg) were phosphorylated by commercial rat protein kinase CK2 (Promega), human protein kinase A (PKA) (Millipore), rat protein kinase C (PKC) (Promega) or by using *S. mansoni* total protein extract as a source of kinases. Reactions were carried out in CK2 buffer (25 mM Tris-HCl, pH 7.4, 200 mM NaCl, 10 mM MgCl_2_, and 0.1 mM ATP), at 37°C at different times, PKA buffer (100 mM HEPES, pH 7.0, KCl 200 mM , 20 mM MgCl_2_, 0,1 mM ATP) and PKC buffer (30 mM Tris-HCl, pH 7.6, 2 mM dithiothreitol, 6 mM Mg(CH_3_COO)_2_, 0.4 mM CaCl_2_, 0,6 µg 1,2-Diacyl-*sn*-glycero-3-phospho-L-serine, 0,12 mM ATP) for one hour at 30°C. Reactions were carried out in the presence of 1 µCi [γ ^32^P]ATP (PerkimElmer). The reaction was stopped by adding SDS-PAGE sample buffer (50 mM Tris-HCl pH 6.8, 2% SDS, 0.1% bromophenol blue, 10% glycerol and 100 mM dithiothreitol). For the unphosphorylated control reactions, proteins were incubated in phosphorylation reactions lacking the protein kinase, ATP or buffer. The phosphorylation status of the proteins was examined by autoradiography and protein input controls were examined by Coomassie Blue R-250 staining.

### DNA supercoiling assay

DNA supercoiling assays were carried out as previously described [24]. Briefly, CsCl-purified supercoiled plasmid pTZ19R was relaxed at a DNA concentration ∼170 µg/ml in Topoisomerase I (Topo I) relaxation buffer (50 mM NaCl, 50 mM Tris–HCl, pH 7.5, 1 mM EDTA, 20% glycerol and 1 mM dithiothreitol) in the presence of topo I (2 units/µg DNA; Promega) at 37°C for 90 min. The relaxed DNA (0.5 µg DNA) was then diluted to final 40 mM NaCl, then the same amount of the Topo I was added, followed by the addition of recombinant SmHMGB1 proteins. The 20 µl reactions were allowed to proceed at 37°C for 60 min after which they were terminated by addition of SDS and NaCl to final 1% and 1 M, respectively. DNA was deproteinized by chloroform/isoamyl alcohol (24∶1) extraction in the presence of 0.02% linear polyacrylamide (LPA, SIGMA). Deproteinized DNA was then precipitated with 2.5 volume of ethanol, washed with 70% ethanol, air-dried and finally dissolved in TE buffer. The occourance of DNA topoisomers was analyzed by electrophoresis in 1% agarose gels in 1× TBE buffer at 3 V/cm for 17 h. The gels were stained with 0.5 µg/ml ethidium bromide, distained in water and photographed through a red filter in an UV-transilluminator (Mini-Bis Pro, Bio Imaging Systems).

### T4 DNA ligase-mediated circularization assay

The circularization assay (or bending assay) was carried out as previously described [26]. Briefly, a ^32^P-labeled-66-bp or a ^32^P-labeled-123-bp DNA fragments [29] (1 nM) with cohesive BamHI ends were pre-incubated on ice for 20 min with appropriate amounts of recombinant proteins (50 ng), total (10 µg), nuclear (4 µg) or cytoplasmic (4 µg) adult worm extracts, in 1× T4 DNA ligase buffer (30 mM Tris–HCl, pH 7.8, 10 mM MgCl_2_, 10 mM dithiothreitol, and 0.5 mM ATP; Promega) in a final volume of 20 µl. The DNA was then ligated with T4 DNA ligase (0.6 unit/reaction; Promega) at 30°C for 30 min, and the ligation reactions were terminated by incubation of samples at 65°C for 15 min. Some of the ligation mixtures were digested after termination of ligations with ∼25 units of Exonuclease III (Promega) at 37°C for 30 min. Recombinant SmHMGB1 or protein extracts were pre-incubated in the presence or absence of anti-SmHMGB1 antibody, pre-immune serum or heparin for 30 min at room temperature before ligase reactions. Before electrophoresis, all DNA samples were deproteinized as described in the DNA supercoiling assay. The protein-free DNAs were loaded on pre-run 6% polyacrylamide gels in 0.5× TBE buffer, and finally resolved at 200 V for 2.5 h at 4°C. After electrophoresis, the gels were vacuum-dried and visualized by autoradiography or PhosphorImager STORM 860 (Molecular Dynamics) using Image Quant 5.2 software.

### Cell culture and transfections

For cell work, a heterologous system was chosen since there is no schistosome cell line available until today (recently reviewed by Quack *et al.* [30]. HeLa cells (CCL-2, purchased from ATCC™) were plated on glass coverslips in 24-well dish (80,000 cells/dish) and cultured in RPMI 1640 medium supplemented with 10% fetal bovine serum, in 5% CO_2_ humidified atmosphere. The cells were transiently transfected with 1 µg of pEGFP-SmHMGB1, pEGFP-SmHMGB1-S172A/S174A or empty pEGFP plasmids, using Lipofectamine™ 2000 (Invitrogen). Cells were observed 24 h after transfections and proceeded with the treatment of 100 nM okadaic acid (OA) for 6 h to inhibit protein phosphatases and thus, enhance phosphorylation. Another batch of cells was pre-treated with 75 µM 4,5,6,7- tetrabromobenzotriazole (TBBt, a specific CK2 inhibitor) [31] for 1 h and then treated with OA (as above). Controls included cells expressing EGFP-SmHMGB1, EGFP-SmHMGB1-S172A/S174A or empty EGFP, without any treatment. Cell viability was assayed by measuring LDH activity (CytoTox 96, Promega) and by Trypan Blue staining.

### EGFP imaging

Cells expressing EGFP-SmHMGB1, EGFP-SmHMGB1-S172A/S174A or EGFP alone, treated with TBBt and/or OA, and non-treated controls were fixed in 4% Paraformaldehyde for 1 h at room temperature. After fixation, cells were washed with PBS 1×. Nuclei were stained with DAPI for 5 min at room temperature. Images were taken on a Zeiss Axio Observer.Z1 invert microscope equipped with 100× objective lens and an AxioCam MRm camera, in the ApoTome mode.

### Transmission Electron Microscopy and Immunolabeling

*S. mansoni* male-adult worms were obtained from saline hepatic perfusion, fixed in 0.7% glutaraldehyde (v/v), 0.1% picric acid, 1% sucrose, 2% paraformaldehyde, and and 5 mM CaCl_2_ in 0.1 M cacodylate buffer (pH 7.2), dehydrated in ethanol and embedded in Unicryl (Ted Pella, Redding, CA). Ultrathin sections were quenched in 50 mM NH_4_Cl for 30 min and incubated in the presence of polyclonal anti-SmHMGB1 antibodies. After several washes in PBS 1× and 1% BSA, sections were incubated in the presence of 10 nm of gold-labeled goat anti-rabbit IgG (BB International, UK), washed, and observed in a Zeiss 900 electron microscope. Negative control sections were performed using pre-immune serum (Figure S2).

### Western blotting

Total extract of adult paired-worms was generated by tissue homogenization with PBS 1× in the presence of a protease inhibitor cocktail (SIGMA) followed by 14.000× g centrifugation. Supernatant was collected and contained total soluble proteins. The cytoplasmic and nuclear fractions from 100 couples of adult worms were separated using Cell Lytic™ Nuclear™ Extraction Kit (SIGMA). Briefly, the worms were washed in cold PBS 1×, incubated in a hypotonic lysis buffer (100 mM HEPES, pH 7.9, 15 mM MgCl_2_, 100 mM KCl) and centrifuged at 1.000× g. The supernatant (cytoplasmic fraction) was kept in cold and the pellet (nuclear fraction) was resuspended in extraction buffer (20 mM HEPES pH 7.9, 1.5 mM MgCl_2_, 0.42 M NaCl, 0.2 mM EDTA and 25% (v/v) glycerol). The nuclear fraction was obtained by 20.000× g centrifugation. Protein concentration was determined by the Bio-Rad Protein Assay (Bio-Rad). Western blot analysis was carried out using polyclonal anti-SmHMGB1 or anti-acetylated histones (a kind gift of Dr. Cristina Motta, Instituto de Biofísica, UFRJ), and a HRP-labeled anti-rabbit as the secondary antibody. Blots were developed with ECL enhanced chemiluminescent reagents (Pierce).

### Immunoprecipitation

Total extract of adult worms was generated as described above. Nuclear and cytoplasmic fractions were pre-cleared by incubation with protein A/G-Sepharose (Santa Cruz) at 4°C for 30 min. The pre-cleared extracts (500 µg) were incubated with rabbit polyclonal anti-SmHMGB1 for 15 h at 4°C and then protein A/G-Sepharose was added and incubated for 3 h at 4°C. Immuneprecipitated complexes were collected by centrifugation and extensive washed with PBS 1×. Collected complexes were fractionated by 12% SDS-PAGE, blotted to membranes, and detected by rabbit polyclonal anti-phosphoserine antibodies (Chemicon).

### Immunohistochemistry

Livers from 60 day-*S.mansoni* infected Swiss mice were immediately embedded in OCT medium in a pre-cooled breaker of isopentene and frozen in liquid N_2_. Seven micrometer cryostat sections were adhered to glass slides and fixed in cold acetone for 30 minutes at −20°C. The sections were washed three times with PBS 1×, 0.03% Triton X-100 and blocked with PBS 1× and 5% BSA for 30 minutes. The sections were incubated with polyclonal anti-SmHMGB1 and/or monoclonal anti-phosphoserine (SIGMA) in PBS 1× for 15 h at 4°C. After washing, an Alexa Fluor 488 conjugated anti-rabbit (Invitrogen) and/or an Alexa Fluor 555 conjugated anti-mouse (Invitrogen) were added for 1 h at room temperature. Negative controls were obtained using only secondary antibodies. The sections were mounted in prolong® Gold antifade reagent with DAPI (Invitrogen). All experiments were repeated three times, and representative images were taken by a Leica TCS SP5 AOBS confocal microscope.

## Results

### *In vitro* analysis of SmHMGB1 phosphorylation

**Figure 1:**
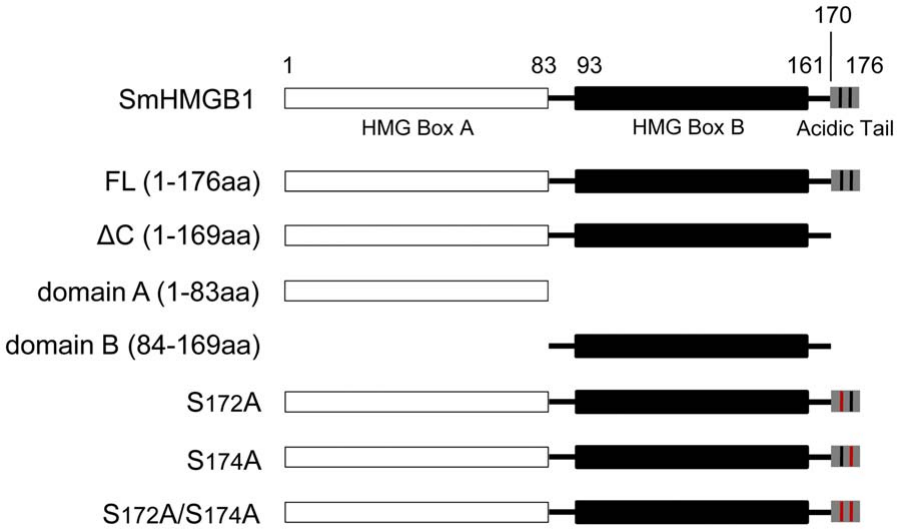
Schematic diagram of the *Schistosoma mansoni* HMGB1 gene structure and constructed mutants. SmHMGB1 full length (FL) consists of two DNA-binding domains, the HMG box A (aa 1–83), HMG box B (aa 84–169) and a short acidic C-terminal domain (170–176). ΔC (aa 1–169) refers to SmHMGB1 lacking only its acidic C-terminal domain; domain A (aa 1–83) refers to SmHMGB1 lacking its HMG box B domain; domain B (aa 84–169) refers to SmHMGB1 lacking its HMG box A domain; S172A refers to SmHMGB1 with a point mutation at serine 172, substituted by alanine; S174A refers to SmHMGB1 with a point mutation at serine 174, substituted by alanine; S172A/S174A refers to SmHMGB1 with two point mutations at serines 172 and 174, both substituted by alanine.

**Figure 2:**
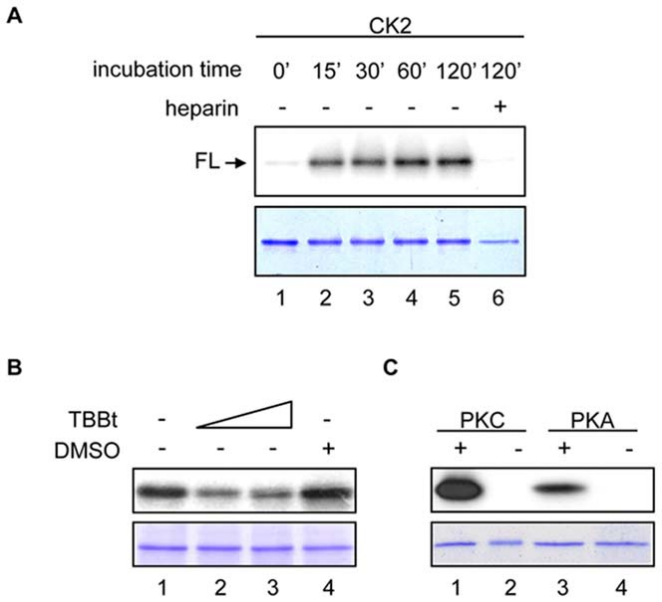
*In vitro* kinase assay of SmHMGB1 phosphorylation. (A) One µg of the recombinant SmHMGB1 full length protein (FL) was used as a substrate for commercial CK2, at various incubation times, in the presence of [γ ^32^P]ATP. Heparin was included to show specific inhibition of CK2. Phosphorylations were analyzed by 15% SDS-PAGE and autoradiography (top panel). Bottom panel is the Coomassie blue stained gel of the SmHMGB1-FL used in the reactions; (B) *S. mansoni* adult worm total protein extract (4 µg) was used, as a source of kinases, in *in vitro* phosphorylation reactions. One µg of recombinant SmHMGB1-FL was incubated with the extract for 1 h in the presence of [γ ^32^P]ATP, and with (1.8 and 3.6 µM) or without TBBt, a specific CK2 inhibitor. TBBt was dissolved in DMSO and we used it as control. Top panel: phosphorylation; bottom panel: Coomassie blue staining; (C) One µg of recombinant SmHMGB1 full length protein (FL) was used as a substrate for commercial PKA and PKC in phosphorylation reactions for 1 h in the presence of γ[^32^P]ATP. These experiments were repeated three times.

**Figure 3:**
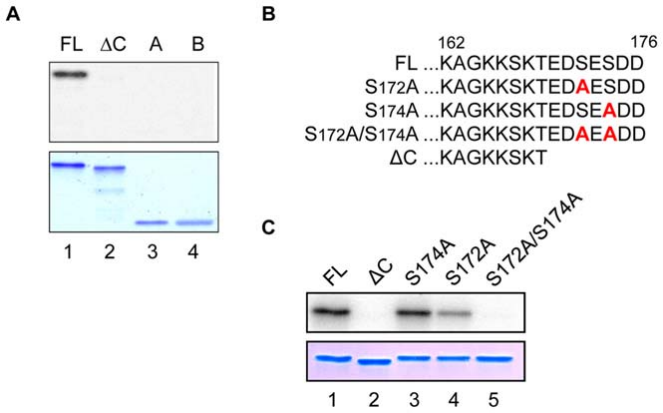
Mapping of CK2 phosphorylation sites in SmHMGB1. (A) One µg of the recombinant SmHMGB1-FL, SmHMGB1-ΔC, SmHMGB1-domain A and SmHMGB1-domain B were assayed for CK2 phosphorylation, as described in Figure 2. Top panel shows phosphorylation of SmHMGB1-FL (lane 1); no phosphoprylation was observed with the deleted constructions (lanes 2–4). Bottom panel shows the Coomassie blue staining of the purified recombinant proteins used in the phosphorylation assay. (B) Only part of the protein is represented (from aa 162 to the end of the protein) to show where the point mutations took place. The two serine residues located at the acidic C-terminal tail of SmHMGB1-FL were substituted to alanines (in red), accordingly. (C) One µg of the recombinant SmHMGB1-FL, SmHMGB1-ΔC or mutated constructs were assayed for CK2 phosphorylation. These experiments were repeated three times.

HMGB1 from different organisms (mammalian, insects and plants) have been shown to be substrates for different kinases (CaMK, PKC, cdc2, CK2) [16]–[19]. Since these phosphorylations proved to be important for the biological functions of these different HMGB1 proteins, we asked the question whether SmHMGB1 could also be phosphorylated. First, we subjected the full amino acid sequence of SmHMGB1 to an *in silico* analysis using the software NetPhosK 1.0 server (http://www.cbs.dtu.dk/services/NetPhosK). The program revealed putative phosphorylation sites for CK2, PKC and PKA. The CK2 putative sites were identified at positions S167, T169, S172 and S174 and we used an *in vitro* biochemical approach to identify the actual CK2 phosphorylation site(s) of SmHMGB1. We performed phosphorylation reactions with recombinant full length SmHMGB1 (FL), but also assayed other SmHMGB1 gene constructions (Figure 1), individually (see below). We showed that SmHMGB1-FL was a specific substrate for commercial CK2 (Figure 2A). Specificity was demonstrated by addition of heparin, a well-known inhibitor of CK2, which completely abolished SmHMGB1 phosphorylation (Figure 2A, lane 6). Moreover, the use of a synthetic specific inhibitor of CK2, tetrabromobenzotriazole, TBBt, completely abolished phosphorylation of SmHMGB1 (not shown). Since CK2, PKA and PKC of *S. mansoni* were identified in the genome and transcriptome database of the parasite, we assumed that the parasite total protein extract could be a good source of endogenous kinases. We then tested the capacity of this extract to phosphorylate SmHMGB1. In fact, we were able to show that endogenous kinases present in the extract of adult worms were able to phosphorylate the recombinant SmHMGB1-FL (Figure 2B). To evaluate the participation of the endogenous schistosome CK2 in this process, we made use of the CK2 specific inhibitor, TBBt (we avoided using heparin because being a polyanion it could be sequestered by positive molecules present in the extract). TBBt was able to consistently inhibit to 43% (quantified by ImageJ-NIH Software) the phosphorylation of SmHMGB1 (Figure 2B, lanes 2 and 3). Knowing that TBBt inhibited phopshorylation by CK2 only, we assumed that other kinases could be active in the extract of *S. mansoni*. Considering the results from the NetPhosK program, that also identified putative sites for PKC and PKA, we tested the ability of these two enzymes to phosphorylate recombinant SmHMGB1-FL. The results showed that commercial PKC and PKA were able to phosphorylate SmHMGB1-FL (Figure 2C, lanes 1 and 3). When we tested several concentrations of PKC (Bisindolylmaleimide II, Calbiochem) or PKA (H89, LC Laboratories) inhibitors in reactions containing commercial PKC, PKA or the total protein extract (as source of endogenous kinases) of the parasite, inhibition of phosphorylation of recombinant SmHMGB1 were discrete (data not shown). Although it is not clear to us why these inhibitions were weak, it is relevant to point out that commercially available inhibitors of PKA or PKC have been previously described to be somewhat inefficient and/or non-specific in some biological systems [18], [32]. We next wanted to determine what regions or domains of SmHMGB1 were being targeted by CK2. For these phosphorylation experiments, we expressed the first four gene constructs depicted in Figure 1 (recombinant His_6_-tagged SmHMGB1 proteins: full length [FL], the protein lacking its acidic C-terminal tail [ΔC], the HMG box domain A [domain A] and the HMG box domain B [domain B]). We showed that only the full length protein was phosphorylated by CK2 (Figure 3A, lane 1). Importantly, the protein construct lacking its acidic C-terminal tail (see Figure 1, ΔC and Figure 3B, ΔC) failed to be phosphorylated (lane 2). This result indicated to us that the phosphorylation site (s) of CK2 was (were) localized within the seven acidic residues contained in the acidic tail of SmHMGB1 (aa 170–176, see Figure 1, FL).

Protein kinase CK2 phosphorylates serine and/or threonine residues that are embedded around negatively charged amino acids. The acidic tail of SmHMGB1 contains two serine residues surrounded by three aspartic acid and two glutamic acid residues (see Figure 3B, FL). This observation prompted us to introduce point mutations at these two serine residues (see Figure 3B, S172A, S174A and S172A/S174A) and perform phosphorylation reactions using these mutants as substrates for CK2. The results obtained with the double mutant (S172A/S174A) showed that the two serine residues present in the acidic C-terminal tail of SmHMGB1 were indeed the phosphorylation sites for CK2 (Figure 3C, lane 5). Phosphorylation reactions using the mutant S172A consistently revealed a slight decrease in phosphorylation signal when compared with the FL phosphorylation (Figure 3C; compare lane 1 with lane 4). The weaker CK2-phosphorylation of S172A could be due to partial conformational constraint of this specific mutant.

### DNA binding activities of phosphorylated SmHMGB1

**Figure 4:**
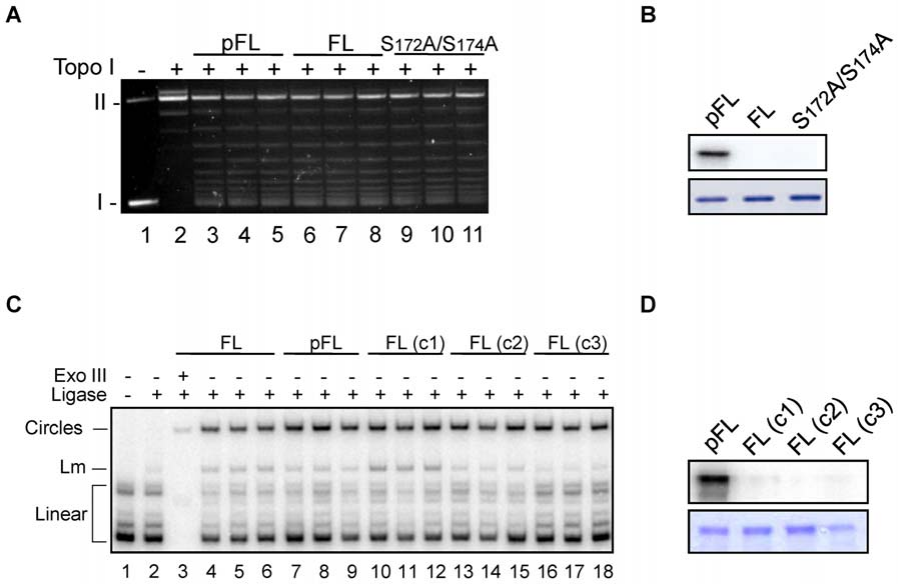
DNA supercoiling and bending assays by phosphorylated SmHMGB1. (A) Circular relaxed plasmid pTZ19R DNA was incubated in the presence of topoisomerase I with 1 µg of recombinant SmHMGB1-FL or SmHMGB1-S172A/S174A that were phosphorylated (lanes 3–5) or not (lanes 6–8 and 9–11), by CK2. Deproteinized DNA topoisomers were resolved on 1% agarose gels, followed by staining of the gels with ethidium bromide. Form I, supercoiled DNA; form II, relaxed circular DNA. (B) Top panel: autoradiography; bottom panel: Coomassie staining. (C) A ^32^P-labeled 123-bp DNA fragment (∼1 nM) was pre-incubated with 50 ng of recombinant proteins, that were phosphorylated (lanes 7–9) or not (lanes 4–6, 10–12, 13–15 and 16–18), followed by ligation with T4 DNA ligase. Exonuclease III was used to verify the identity of DNA circles. The deproteinized DNA ligation products were subjected to electrophoresis on 6% non-denaturing polyacrylamide gels and visualized by autoradiography. Controls are as follows: FL(c1): SmHMGB1-FL without CK2; FL(c2): SmHMGB1-FL without phosphate; FL(c3): SmHMGB1-FL without CK2 buffer. Linear: linear DNA; Lm: linear multimers. (D) Top panel: autoradiography; bottom panel: Coomassie staining. These experiments were repeated four times.

Differently from canonical transcription factors, HMGB1 proteins do not exhibit DNA-sequence specificity. Alternatively, HMGB1 exhibits a remarkably high affinity for distorted DNA conformations such as supercoiled DNA, four-way junction DNA and DNA bulges, but it can also actively distort DNA by bending or supercoiling and changing of DNA topology [5]. In this work, we used two well-established DNA assays for HMGB1 proteins (supercoiling assay and T4 DNA ligase-mediated circularization assay; see legend for details of the technique), to determine whether phosphorylation influences SmHMGB1 DNA transactions. Our data consistently showed that the supercoiling activities of SmHMGB1 that was not phosphorylated (FL) or SmHMGB1 that was phosphorylated (pFL) by CK2 were basically the same (Figure 4A; compare lanes 3–5 with 6–8). When we used the double mutant in the supercoiling experiment (they were also submitted to phosphorylation), no difference was observed (compare lanes 3–5 with 9–11). The inclusion of the mutants in this experiment aimed at certifying that, even though they would not be phosphorylated, the amino acid substitutions themselves would not alter the functionality of the protein. Similarly with the supercoiling assay, when we performed DNA bending assays using either a 123 bp-DNA fragment (Figure 4C) or a 66 bp-DNA fragment (data not shown), the formation of minicircles were not affected by phosphorylated SmHMGB1 (Figure 4C, compare lanes 7–9 with lanes 10–18, which are controls lacking components of the phosphorylation reaction). Recombinant SmHMGB1 proteins were tested for their integrity and activity before being submitted to phopshorylation reactions (lanes 4–6), see figure legend for details (panels B and D are controls showing that the SmHMGB1 proteins that were used in the reactions were phosphorylated [pFL] or not [FL]). A pixel quantification of the bands were performed and confirmed that no significant differences were observed between phosphorylated (lanes 7–9) and non-phosphorylated (lanes 10–18) SmHMGB1 (data not shown).

### Effect of phosphorylation in the nucleocytoplasmic shuttling of SmHMGB1

**Figure 5:**
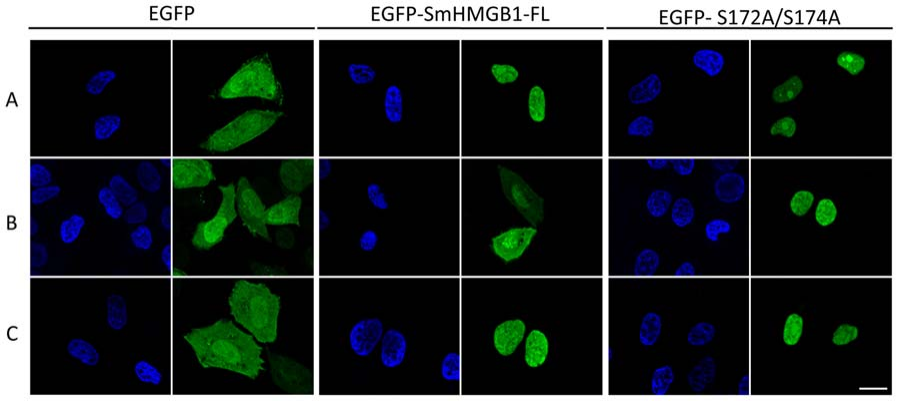
Phosphorylation of SmHMGB1 mediates its cellular traffic in HeLa cells. HeLa cells were transfected with empty control plasmid pEGFP, pEGFP-SmHMGB1-FL or pEGFP-SmHMGB1-S172A/S174A plasmids and untreated (panels A) or treated with 100 nM okadaic acid (OA) for 6 h (panels B) or with OA+75 µM TBBt (panels C). SmHMGB1-EGFP fusion proteins were detected by fluorescence microscopy. Nuclei were stained with DAPI. Cell viability was assessed by Trypan blue and LDH activity (data not shown). Scale bar 3 µm. This result is a representative of four independent experiments.

To investigate whether phosphorylation of SmHMGB1 influences its nuclear transport *in vivo*, we made use of a mammalian heterologous system (remember that heterologous cells had to be used since a schistosome cell line is not yet available [30]. In Figure 5, HeLa cells were transiently transfected with either EGFP plasmid alone, EGFP-SmHMGB1 or EGFP-SmHMGB1-S172/174A, and received treatments of okadaic acid (OA, a protein phosphatase inhibitor that enhances phosphorylation) or TBBt (a specific inhibitor of CK2).

Control cells that were transfected with EGFP plasmid alone, not treated or treated with OA or/and TBBt revealed the presence of EGFP either in the nucleus or in the cytoplasm (Figure 5, panels A, B and C). Transfected cells that were not treated with OA revealed the presence of EGFP-SmHMGB1 exclusively in the nucleus (Figure 5, panel A). Treatment of transfected cells with OA resulted in a significant translocation of nuclear EGFP-SmHMGB1 to the cytoplasm (Figure 5, panel B). When transfected cells were treated with TBBt prior to addition of OA, no EGFP-SmHMGB1 was observed in the cytoplasm (Figure 5, panel C), indicating that CK2 phosphorylation played an important role in the traffic of SmHMGB1 from the nucleus to the cytoplasm. Cells that were transfected with EGFP-SmHMGB1-S172A/S174A (the construct that contains the mutations at the two serine residues) but that received no treatment, showed localization of the protein exclusively in the nuclei (Figure 5, panel A). When these transfected cells were treated with OA (panel B) or OA plus TBBt (panel C), no cytoplasmic translocation was observed whatsoever, showing that the CK2-phosphorylation sites of SmHMGB1 are important mediators for the protein translocation.

### Presence of native SmHMGB1 in the nucleus and cytoplasm of adult *S. mansoni* cells

**Figure 6:**
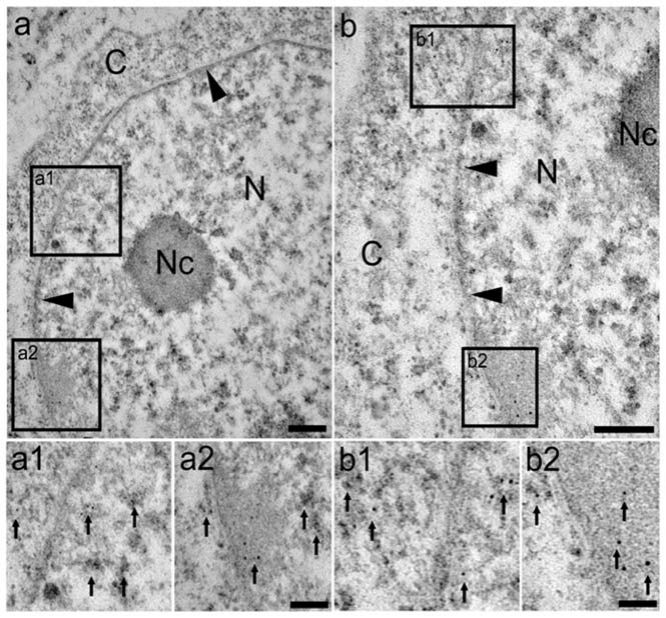
*In situ* localization of native SmHMGB1 protein in the nucleus and cytoplasm of *S. mansoni* cells. (a and b) Transmission electron microscopy (TEM) of cells from *S. mansoni* male adult worms showing the nucleus (N), nucleolus (Nc), cytoplasm (C) and the nuclear membrane (arrowheads). Insets a1, a2, b1 and b2 depict a closer visualization of the interface between the nucleus and the cytoplasm. The immunogold staining shows the labeling of SmHMGB1 in both cellular compartments (arrows indicate representative SmHMGB1 labeling). Bars: a and b = 100 nm; a1 and a2 = 50 nm. This image is a representative of several cells observed under TEM.

We have shown (figure 5) that SmHMGB1 can traffic between the nucleus and cytoplasm of mammalian cells. In order to determine whether SmHMGB1 can be found in these two cellular compartments of *S. mansoni* cells, we used transmission electron microscopy and immunolabeling of ultra-thin sections of male adult worms (Figure 6). The electron microscopy of a male worm cell depicts clearly the nucleus (N), nucleolus (Nc), and cytoplasm (C). The nuclear membrane (arrowheads) is also registered (Figure 6, panels a and b). Boxes a1, a2, b1 and b2 show at a higher magnification the intense immunogold labeling of SmHMGB1. The arrows indicate the presence of endogenous SmHMGB1 in the nucleus, on the nuclear membrane and in the cytoplasm of a schistosome cell. No labeling was observed (control) when sections were performed with pre-immune serum (Figure S2).

### Active and phosphorylated SmHMGB1 in the cytosolic fraction of *S. mansoni* cells

**Figure 7:**
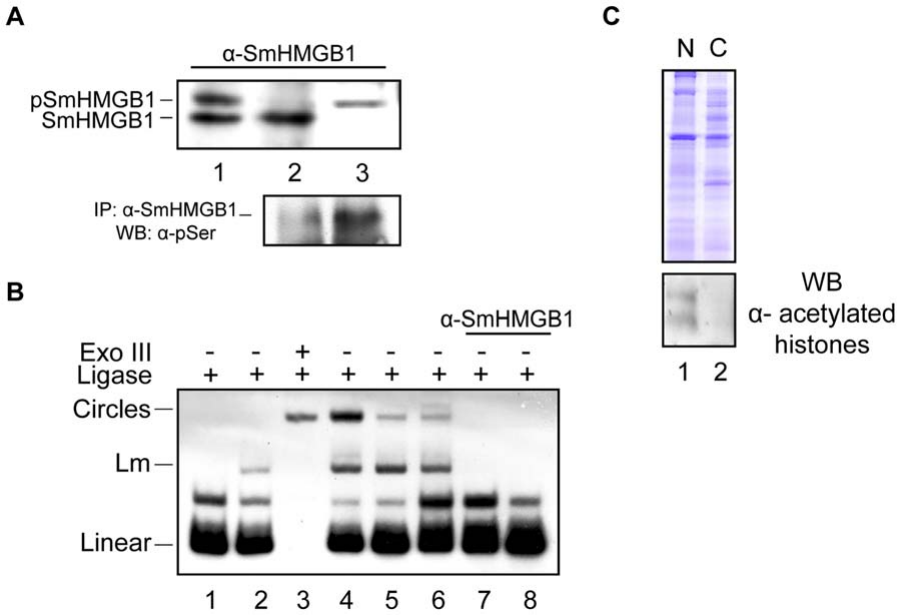
Endogenous phosphorylation of SmHMGB1 did not alter its DNA bending activity. (A) Western blot analysis was carried out with *S. mansoni* protein extracts and an anti-SmHMGB1 polyclonal antibody. SmHMGB1 antibodies detected two proteins in the total extract (top panel, lane 1). The lower molecular weight protein was detected in the nuclear extract only (top panel, lane 2); the higher molecular weight protein was detected in the cytosolic extract only (top panel, lane 3). SmHMGB1 that was immunopreciptated from the nuclear or cytosolic extracts using SmHMGB1 antibodies were reacted against anti-phosphoserine antibodies in a Western blot (bottom panel, lane 2 (nuclear extract) and 3 (cytosolic extract); phosphorylation of SmHMGB1 at serines is indicated by pSmHMGB1). (B) Bending assay: a ^32^P-labeled 123-bp DNA fragment (1 nM) was pre-incubated with 10 µg of total (lane 4), 4 µg of nuclear (lane 5) or 4 µg of cytosolic (lane 6) protein extracts from *S. mansoni* adult worms, and the assay performed as described in Figure 4C. To make sure that the circles were formed by the activity of SmHMGB1, nuclear or cytosolic (lanes 7 and 8, respectively) extracts were incubated with anti-SmHMGB1 antibodies prior bending reactions. Extracts that were incubated with SmHMGB1 antibody did not show any circularization (or bending) activity (lanes 7 and 8). The Exo III control proved the identity of the circles. (C) Top panel: SDS-PAGE of *S. mansoni* adult worm nuclear (N) or cytosolic (C) extracts. Bottom panel: Western blot with anti-acetylated histones, showing no cross-contamination between the two extracts. These experiments were repeated three times.

Besides electron microscopy, we used biochemical approaches with protein extracts from adult worms to determine if SmHMGB1 is endogenously phosphorylated. When we reacted the total extract of *S. mansoni* against SmHMGB1 antibody, two bands with slightly differences in size were consistently detected (Figure 7A, top panel, lane 1). When the nuclear extract was reacted against SmHMGB1 antibody, only the lower molecular weight band was detected (Figure 7A, top panel, lane 2). However, when the cytosolic extract was reacted against the same antibody, only the higher molecular band was detected (Figure 7A, top panel, lane 3). These results prompted us to test whether this slight difference in protein size found only in the cytosolic fraction could be due to phosphorylation. When we immunoprecipitated SmHMGB1 proteins from the nuclear or cytosolic extracts and then reacted them against an anti-phosphoserine antibody, we observed that the cytosolic SmHMGB1 was highly phosphorylated (Figure 7A, bottom panel, lane 3). Alternatively, SmHMGB1 that was present in the nucleus showed only residual phosphorylation (Figure 7A, bottom panel, lane 2).

Since we showed that phosphorylation of recombinant SmHMGB1 did not interfere with its DNA binding activity (Figure 4), we then wanted to test if phosphorylation of endogenous SmHMGB1 would behave similarly. For this, we performed the T4 DNA ligase-mediated circularization assay (or DNA bending assay) using *S. mansoni* total, nuclear or cytosolic extracts as source of phosphorylated or non-phosphorylated SmHMGB1 (Figure 7B). When we used the total extract, a significant formation of circles (DNA bending) was observed (Figure 7B, lane 4). When the nuclear or cytosolic extracts were used, the formation of circles was also observed (Figure 7B, lanes 5 and 6). It is important to point out that the activity of the nuclear SmHMGB1 (presumably not phosphorylated) or cytosolic SmHMGB1 (presumably phosphorylated) were comparable. We were able to prove that the formation of the circles was a result of the activity of the nuclear or cytosolic SmHMGB1 by the addition of an anti-SmHMGB1 antibody prior to the bending reaction, where no circles were formed (Figure 7B, lanes 7 and 8). To further confirm that SmHMGB1 antibody was not unspecifically targeting the ligase reaction, we carried out an additional DNA bending reactions containing SmHMGB1 specific antibody, heparin (which can sequester HMGB1) or the pre-immune serum (Figure S1, see legend for details).

The formation of circles was determined by the treatment of Exonuclease III (Exo III), an enzyme that degrades only linear DNA, but not circular DNA. Linear multimers (Lm) were also observed (HMGB1 proteins are known to enhance the formation of linear multimers by the T4 DNA ligase) [33].

To certify that the cytosolic extract was not contaminated with nuclear proteins, we performed a Western blot (Figure 7C) with *S. mansoni* nuclear or cytosolic extracts and reacted them against anti-acetylated histone antibodies. Figure 7C shows that histones were only detected in the nuclear extract.

### Phosphorylated SmHMGB1 is amply distributed in schistosomotic granuloma

**Figure 8:**
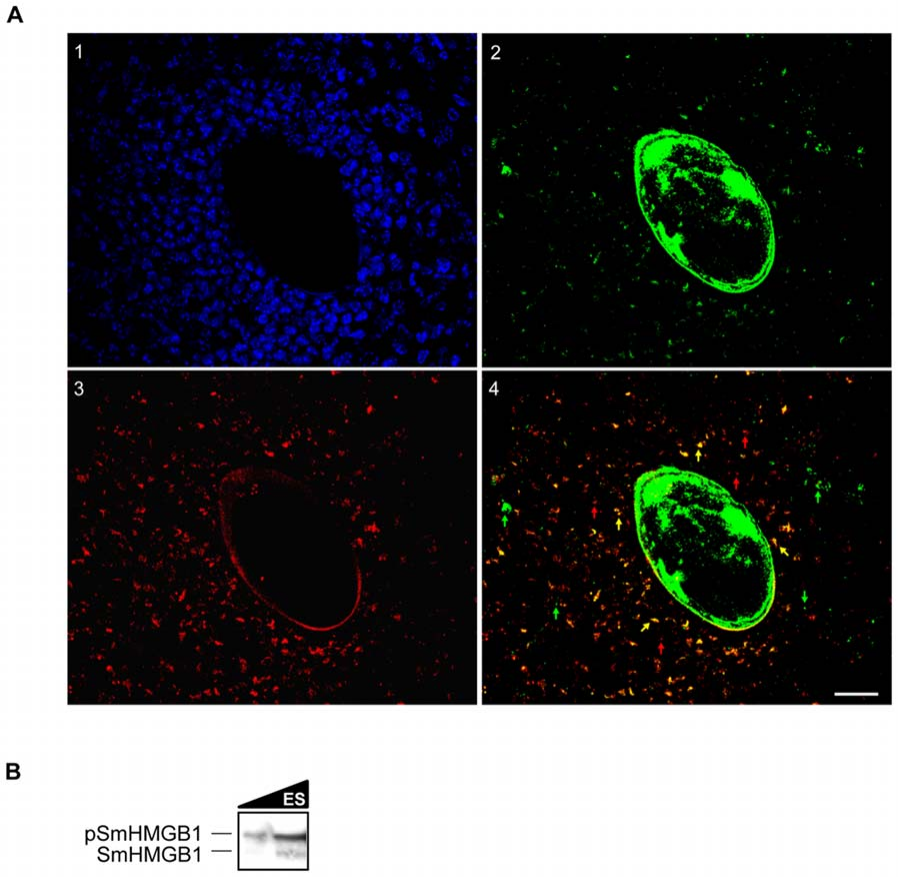
Localization of phosphorylated SmHMGB1 in the granulomatous liver. (A) Immunostaining of hepatic granuloma with a *S. mansoni* egg in the center. Nuclei were stained with DAPI (1); Detection of SmHMGB1 using an anti-SmHMGB1 polyclonal antibody (2). Detection of phosphorylated SmHMGB1 using an anti-phosphoserine monoclonal antibody (3). A significant amount of secreted SmHMGB1 detected in the granulomatous liver is phosphorylated (merged images of panels 2 and 3). In panel 4, green arrows point to secreted but non-phosphorylated SmHMGB1; red arrows point to phosphorylated proteins from the host; orange arrows point to secreted phosphorylated SmHMGB1. Controls with the pre-immune sera (not shown) or with the secondary antibody only (Figure S3), exhibited a residual auto-fluorescence from the eggshell. Scale bar: 20 µm. This figure is a representative of the several egg-induced granuloma analyzed from three independent mice livers. (B) Eggs (∼10^6^ eggs) from these livers were processed and egg secretions (ES) were assayed by Western blot using anti-SmHMGB1 antibody. The two isoforms (phosphorylated and unphosphorylated) of SmHMGB1 were detected in egg secretions. However, the high molecular weight (phosphorylated) isoform is significantly more abundant in egg secretions (top band).

Considering the role of phosphorylation in the extracellular release of mammalian HMGB1 as well as the pro-inflammatory activity of the protein in modulating the pathogenesis of several inflammatory diseases, we next evaluated the phosphorylation status of SmHMGB1 proteins that were secreted and lodged in the schistosomotic liver granuloma. By immunofluorescence assays using an anti- SmHMGB1 antibody, we were able to demonstrate the presence of a significant amount of SmHMGB1 in the eggshell and a considerable amount of egg-released SmHMGB1 throughout the granulomatous liver tissue (Figure 8, panel 2). We next used anti-phosphoserine antibodies and showed that a number of proteins present in the host granulomatous liver were phosphorylated (Figure 8, panel 3). Co-localization analysis confirmed that a large amount of egg-secreted SmHMGB1 proteins were phosphorylated (Figure 8, panel 4, merge of 2 and 3, orange arrows). Immunoreaction with the secondary antibody only, revealed the previously described auto-fluorescence of the eggshell [34] (Figure 8, panels 2, 3 and 4; Figure S3). Molecules that were red-labeled only (in panel 3, and red arrows in panel 4), likely represent serine-phosphorylated proteins from the host and possible from the schistosome eggs. However, a smaller proportion of the SmHMGB1 proteins that were not modified by phosphorylation were identified in the granulomatous liver tissue (green arrows in panel 4). It is possible that this population of SmHMGB1 proteins (green fluorescence only) could have reached the extracellular space through other modifications, such as acetylation and/or methylation. Indeed, acetylation of SmHMGB1 plays a role in its cellular exit [28]. In addition, we have shown that SmHMGB1 is arginine-methylated (unpublished results). Importantly, one cannot exclude the possibility that extracellular SmHMGB1 molecules carry multiple modifications. In fact, the interdependence of the post-synthetic acetylation and phosphorylation of mHMGB1 has been reported [35].

In order to evaluate the biochemical profile of extracellular SmHMGB1 proteins located in the granuloma (Figure 8, panel 4), we performed a Western blot analysis and confirmed that egg-secreted SmHMGB1 proteins were modified by phosphorylation (Figure 8B, top band).

## Discussion

HMGB1 proteins have evolved and developed the ability to act both as nuclear factors for the regulation of gene transcription and to contribute to the induction of innate and adaptive immune responses by activating membrane receptor-mediated signal transduction pathways. The combination of transcriptional and extracellular capabilities provides them with the dual capacity of promoting gene expression and mobilizing host defense.

Treatment with inhibitors (anti-HMGB1 antibodies and pharmacological agents) of the mammalian HMGB1 pro-inflammatory activity is beneficial and reduces inflammation in a dozen of preclinical animal studies [9]–[10], [36]–[38]. Therefore, substantial work has been conducted to elucidate the mechanisms by which HMGB1 is released. Current data support an active process initiated by HMGB1-histone disengagement, HMGB1 hyperacetylation, and shuttling of the protein from the nucleus to cytoplasm [6]. Phosphorylation of HMGB1 has also been demonstrated to be essential for this translocation event [16]–[18].

In the present work we aimed to determine the role of phosphorylation of SmHMGB1 in its nucleocytoplasmic shuttling and to correlate its extracellular location to its ability to trigger inflammation.

Despite the overall homology between SmHMGB1 and mHMGB1, their phosphorylation statuses are somewhat distinct. Mammalian HMGB1 has been shown to be phosphorylated by CaMK [17] and PKC [18] and at serine residues located at the two putative NLS, one localized in the HMG box A and the other localized between the HMG box B and the acidic tail [6]. Recently, it has been assumed that serine phosphorylation of mHMGB1 NLS may reduce its DNA-binding and cooperates to its cytoplasmic transport [19]. However, no experiments have been performed by these authors to prove this assumption.

In the case of SmHMGB1, phosphorylation was achieved by PKC, PKA and CK2 (figure 2). CK2 phosphorylation (but not PKC or PKA) was mapped at serines 172 and 174, both located within the short C-terminal acidic tail of the protein (Figure 3). SmHMGB1 contains one putative nuclear localization signal (NLS), at positions (residues) 87 to 90, localized in the linker between the HMG box A and B, and a putative nuclear exportation signal (NES), at positions 101 to 110, localized in the HMG box B domain [26]. By performing DNA-binding assays amply used to test HMGB1 activities, we clearly showed that phosphorylation of either recombinant or native SmHMGB1, did not enhance or reduce their DNA-binding activities (Figure 4 and 7).

Mammalian HMGB1 is a very mobile nuclear protein and the association of mHMGB1 with chromatin is transient [39]. The nucleosome would be visited by mHMGB1 every 2 seconds and the protein would stay there for a small fraction of a second [40]. Thus, mHMGB1 is continuously and rapidly exchanged between cytoplasm and chromosomes. Here, with the results of Figure 6, we showed that under a physiological condition, SmHMGB1 protein was localized in the nucleus, in the nuclear membrane and in the cytoplasm of an adult worm cell, supporting the notion that SmHMGB1 traffics between these two compartments. Additional data from this work suggested that phosphorylation of SmHMGB1 was involved in the shuttle of the protein from the nucleus to the cytoplasm (Figure 5), with its subsequent release to the extracellular space (Figures 8). It is worth to point out that in the case of SmHMGB1, secretion seemed to be dependent of phosphorylation by CK2.

For mHMGB1, it has been shown that the protein was imported to the nucleus by KAP-α1 as a nuclear cargo carrier protein after translation and eventually accumulated in the nucleus [16]. However, a significant fraction of HMGB1 cannot re-enter the nucleus if it has been exported from the nucleus due to phosphorylation [16]. Moreover, inhibition of the exportin protein Crm1 showed a marker reduction of cytoplasmic phosphorylated HMGB1 [19]. Thus, in addition to acetylation, the subcellular localization of mHMGB1 is finely tuned by phosphorylation, although it is still unknown which modification is dominant under physiological conditions.

In the case of SmHMGB1 phosphorylation, while at this moment we can not anticipate which signaling pathway is activated by this modification, with the data presented here we can envision a remarkable role of SmHMGB1 phosphorylation in the modulation of the pathophysiology of schistosomiasis.

In infection with *S. mansoni*, chronic disease is the result of the ongoing host response to accumulating tissue-trapped eggs, with the liver being the principal site affected. Hepatic granuloma are pathogenic because they precipitate fibrosis, which obstructs blood flow, increases portal blood pressure, and ultimately, promotes development of portal-systemic venous shunts [41]. In this work, based on the data that phosphorylated SmHMGB1 is extracellularly released by tissue-trapped eggs, we would like to propose a model where SmHMGB1 can act as a novel egg antigen, promoting inflammation and contributing to granuloma formation.

*Schistosoma mansoni* HMGB1 has been previously shown to be secreted by larvae schistosomula and eggs [27]. *In vitro*, recombinant SmHMGB1 was shown to be a potent inducer of pro-inflammatory cytokines including TNF-α, IL-13, IL-13Rα1, MIP-1α and others [27]. Interestingly, migration of mHMGB1 to organs or tissue sites induced similar pro-inflammatory cytokines, such as TNF-α, IL-1α, IL-1β, IL-1RA, IL-6, IL-8, MIP-1α and MIP-1 β [42].

Tumor necrosis factor-α and IL-13 are believed to provide necessary immune priming for the formation of schistosomotic granuloma [43]–[46]. Although the actual role of TNF-α in schistosomiasis is still debated [47]–[49], several lines of research have implicated this molecule in the chronic form of the disease [50]–[52]. In this regard, TNF-α and HMGB1 have been intimately linked to the pathology of several inflammatory diseases, such as sepsis, rheumatoid arthritis and Crohn's disease [53].

The inflammatory activity of mHMGB1 is dependent upon the oxidation status of the cysteine 106 residue within the HMG box B of the mammalian protein, a region that is critical for stimulating cytokine release and inflammation [10], [54]–[55]. Importantly, the pro-inflammatory activity of SmHMGB1 also appears to be the function of its HMG box B domain [27]. In addition, the cysteine residue 106 is conserved in the SmHMGB1 molecule [26]. A recent study has revealed that cysteine 106 is critically important for mHMGB1 binding to TLR4 [56]. Together, these results indicate that cysteine 106 is required for mHMGB1 (and we believe for SmHMGB1, as well) signaling through TLR4 to stimulate cytokine release and inflammation.

It has been recently demonstrated that the larvae schistosomula tegument activated dendritic cells (DC) to produce IL-12p40, TNF-α and also co-stimulatory molecules CD40 and CD86 through a TLR4-dependent pathway [57]. This finding is especially important because it has been shown that mHMGB1 acts as adjuvant via DC activation, maturation and mobilization [42].

In conclusion, in addition to previously described acetylation, we showed in this study that the subcellular localization and secretion of SmHMGB1 was regulated by phosphorylation. Importantly, we showed that phosphorylated SmHMGB1 was secreted by the eggs that were lodged in the liver of infected mice. We believe that this study will open up a new area of investigation for those interested in understating the pathogenesis of schistosomiais.

## Supporting Information

Figure S1 **DNA bending assay.** A ^32^P-labeled 123 bp-DNA fragment (1 nM) was pre-incubated with 10 µg of total extract from *S. mansoni* adult worms (lanes 3–7), 250 ng of recombinant SmHMGB1 (lanes 8–12). Samples in lanes 5 and 9 were pre-incubated with anti-SmHMGB1 antibody; samples in lanes 6 and 10 were pre-incubated with heparin; samples in lanes 7 and 11 were pre-incubated with the pre-immune serum. The ExoIII control proved the identity of circles (lanes 3 and 12). Two independent experiments showed the same results.
(TIF)

Figure S2 **Negative control of the transmission electron microscopy (TEM) of cells from** ***S. mansoni*** **male adult worms.** No immunogold staining was observed when the pre-immune serum was used. Nucleus (N), nucleolus (Nc) and endoplasmic reticulum (ER). Scale bar 150 nm.
(TIF)

Figure S3 **Auto-fluorescence of** ***S. mansoni*** **eggshell.** Hepatic granuloma with a *S. mansoni* egg in the center was reacted using an Alexa 555 anti-mouse secondary antibody. The auto-fluorescence of *S. mansoni* eggshell is observed. Scale bar: 20 µm.
(TIF)
