# Supplementary figures and images for: CK2 Phosphorylation of Schistosoma mansoni HMGB1 Protein Regulates Its Cellular Traffic and Secretion but Not Its DNA Transactions
Source: PLoS One. 2011 Aug 24;6(8):e23572. doi: 10.1371/journal.pone.0023572 (PMC3160966; doi:10.1371/journal.pone.0023572)

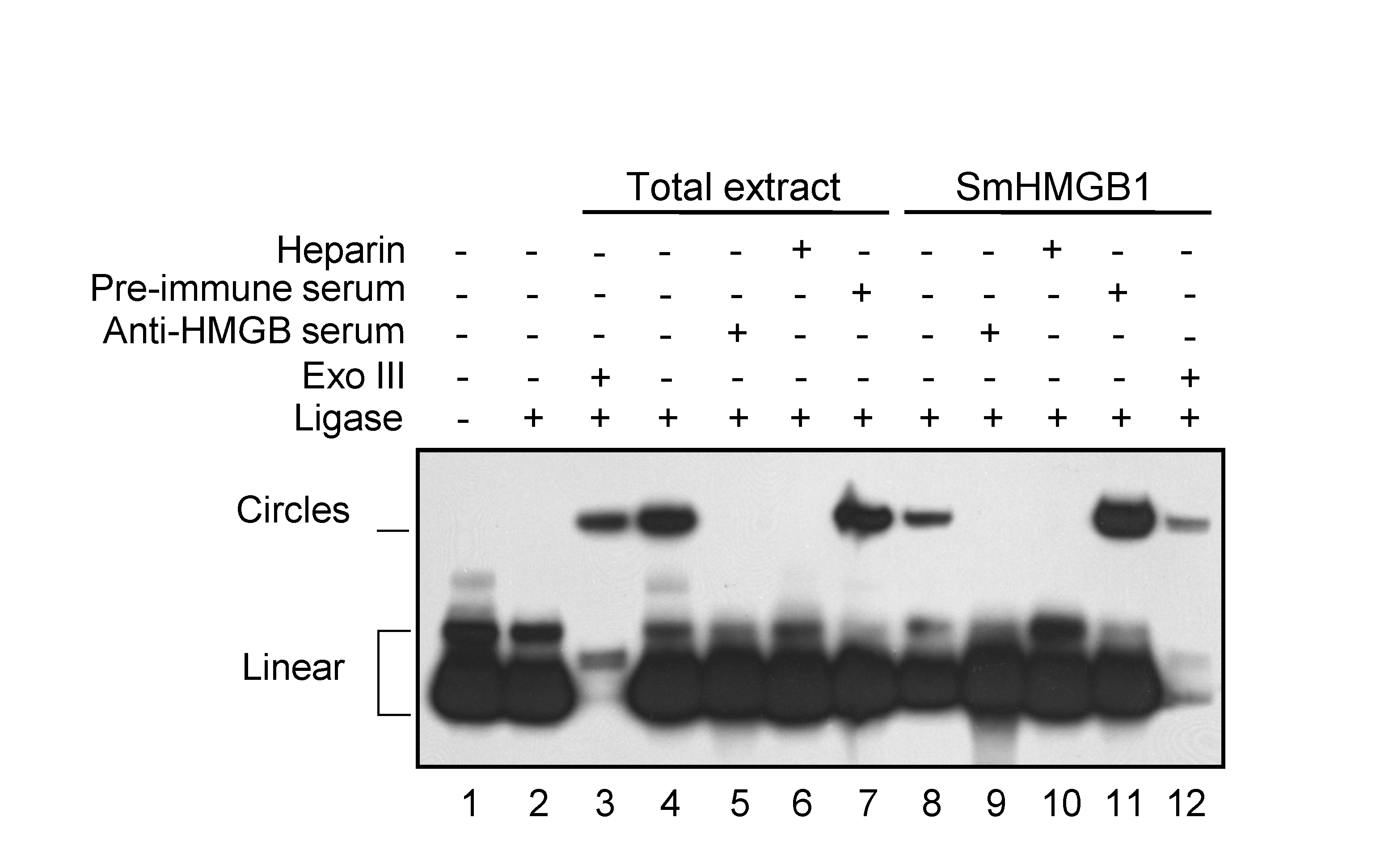

Supplement: Figure S1 — DNA bending assay. A 32P-labeled 123 bp-DNA fragment (1 nM) was pre-incubated with 10 µg of total extract from S. mansoni adult worms (lanes 3–7), 250 ng of recombinant SmHMGB1 (lanes 8–12). Samples in lanes 5 and 9 were pre-incubated with anti-SmHMGB1 antibody; samples in lanes 6 and 10 were pre-incubated with heparin; samples in lanes 7 and 11 were pre-incubated with the pre-immune serum. The ExoIII control proved the identity of circles (lanes 3 and 12). Two independent experiments showed the same results. (TIF) [file pone.0023572.s001.tif]

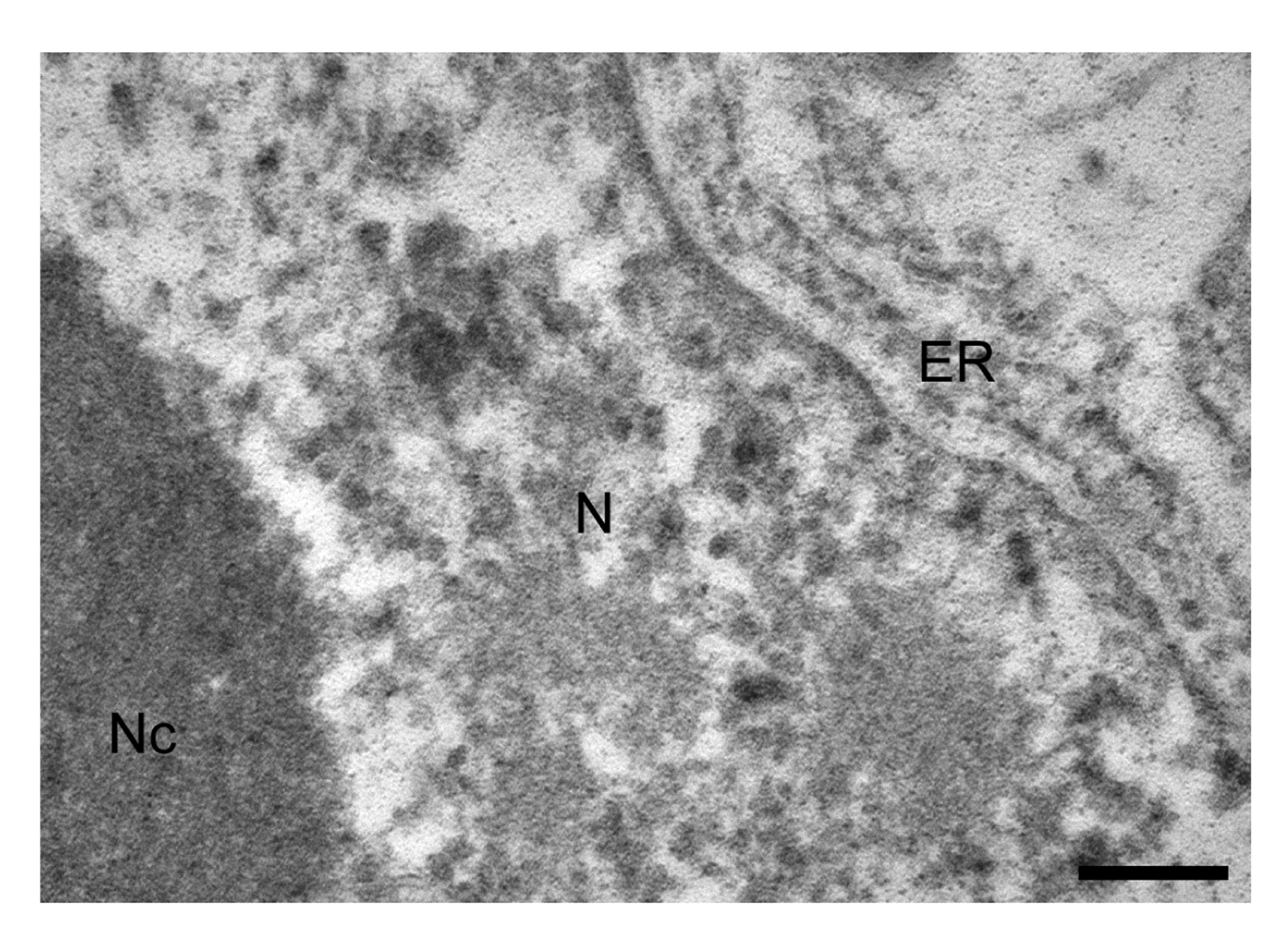

Supplement: Figure S2 — Negative control of the transmission electron microscopy (TEM) of cells from S. mansoni male adult worms. No immunogold staining was observed when the pre-immune serum was used. Nucleus (N), nucleolus (Nc) and endoplasmic reticulum (ER). Scale bar 150 nm. (TIF) [file pone.0023572.s002.tif]

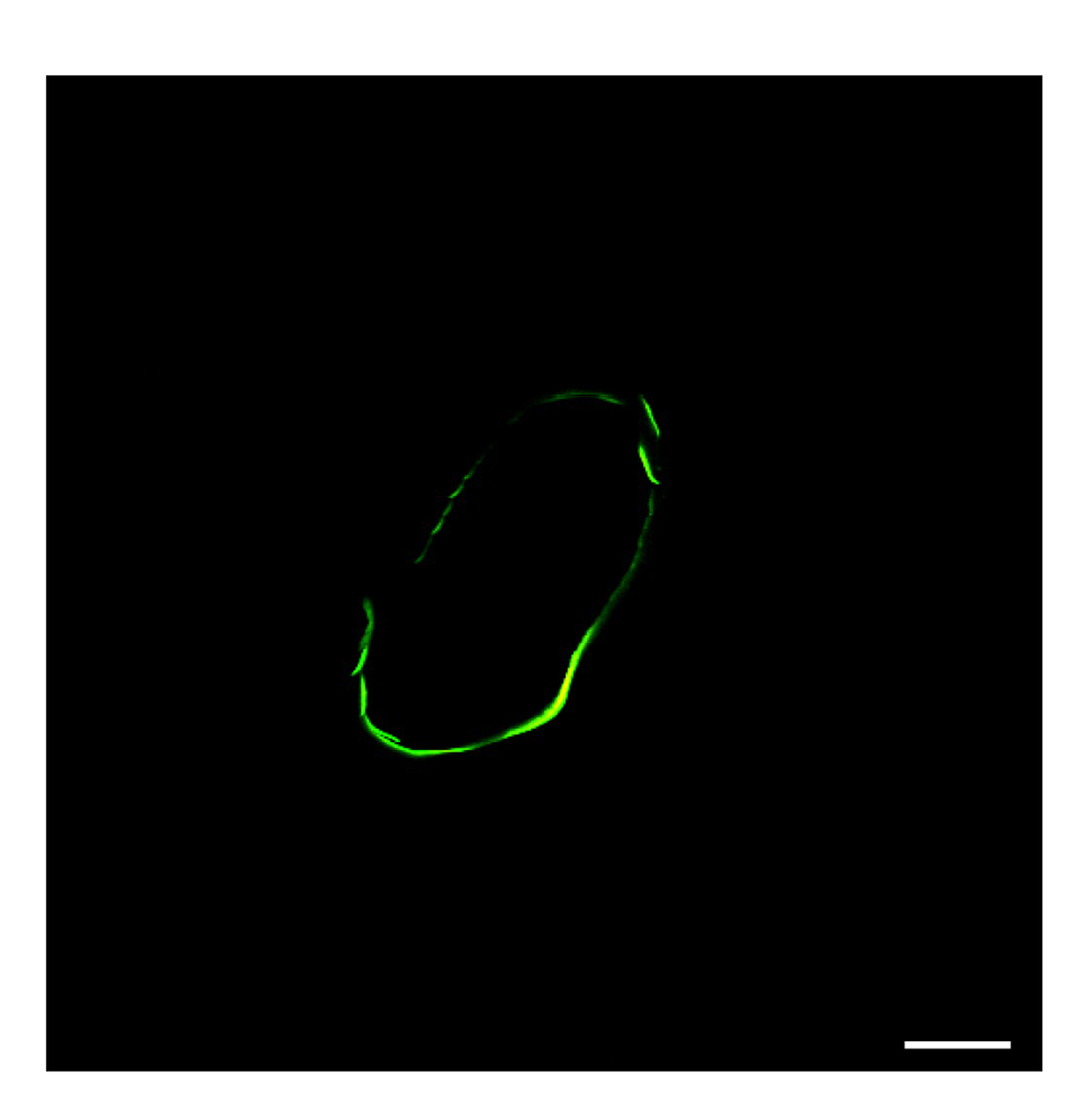

Supplement: Figure S3 — Auto-fluorescence of S. mansoni eggshell. Hepatic granuloma with a S. mansoni egg in the center was reacted using an Alexa 555 anti-mouse secondary antibody. The auto-fluorescence of S. mansoni eggshell is observed. Scale bar: 20 µm. (TIF) [file pone.0023572.s003.tif]
